# Supplementary material for: Strong Genetic Overlaps Between Dimensional and Categorical Models of Bipolar Disorders in a Family Sample
Source: medRxiv. 2024 Mar 26:2023.06.24.23291169. Originally published 2023 Jun 27. Preprint. [Version 3] doi: 10.1101/2023.06.24.23291169 (PMC10327232; doi:10.1101/2023.06.24.23291169)
Supplement: Supplement 2 [file media-2.docx]

**Figure S1.** Bar plot showing the distribution of Mood Disorder Questionnaire (MDQ) scores for the whole sample (n = 726)

**Figure S2**. Plot of the eigenvalues based on the data from the Principal Component Analysis. Eigenvalues are shown as bar plots in blue. Cumulative variability % is shown as a red line. PC = principal component
